# Revolutionizing tumor diagnosis and treatment: the promise of DNA nanotechnology

**DOI:** 10.3389/fimmu.2025.1637539

**Published:** 2025-08-29

**Authors:** Yi Zhou, Ning Wang

**Affiliations:** ^1^ Department of Nuclear Medicine, The First Affiliated Hospital of Hebei North University, Zhangjiakou, Hebei, China; ^2^ Department of Gastroenterology and Hepatology, Tianjin University Central Hospital, Tianjin, China; ^3^ Department of Gastroenterology and Hepatology, The Third Central Hospital of Tianjin, Tianjin, China; ^4^ Tianjin Key Laboratory of Extracorporeal Life Support for Critical Diseases, Tianjin, China; ^5^ Artificial Cell Engineering Technology Research Center, Tianjin, China; ^6^ Tianjin Institute of Hepatobiliary Disease, Tianjin, China

**Keywords:** nanomedicine, DNA nanostructures, aptamer, tumor diagnosis, tumor therapy

## Abstract

Tumors represent a significant challenge to human health, with ongoing difficulties in their diagnosis and treatment. Over recent decades, DNA nanotechnology has emerged as a promising field, demonstrating substantial advancements in drug delivery and disease diagnosis. The inherent biocompatibility and programmability of DNA nanostructures allow for their tailored design and assembly, facilitating the delivery of various therapeutic agents. Due to their ease of modification, these nanostructures can be functionalized to recognize specific targets, enabling the targeted drug delivery and minimizing the adverse effects. Furthermore, DNA nanotechnology contributes to the rapid and sensitive detection of tumor biomarkers, enhancing the early-stage diagnosis of malignant tumors. This article comprehensively reviews advancements in DNA nanomaterial applications for tumor diagnosis and treatment. First of all, in the aspect of tumor diagnosis, this review focuses on the research of DNA nanostructures in the detection of tumor biomarkers, and then introduces the application of DNA nanostructures in tumor therapy, including chemotherapy, gene therapy and immunotherapy. Finally, we summarized the challenges and opportunities of DNA nanomaterials in biomedical research and clinical applications. This review systematically organizes key innovations: (1) first comprehensive analysis of DNA nanostructures design principles for the applications in tumor diagnosis and therapy, and (2) original proposal for overcoming clinical translation barriers through precise design and assembly of DNA nanostructures.

## 1 Introduction

Tumors remain the leading cause of death worldwide, accounting for approximately 19 million new cases and 10 million deaths annually, thereby posing an escalating global challenge (1). As the progression of the increasing and aging global population, tumor-related death has become a huge health and economic burden faced by countries around the world (2, 3). Despite advances in the development of medicine, mitigating the high mortality and poor prognoses associated with tumors remains a critical concern. Over the past few decades, researchers worldwide have been investigating the epidemiology, pathogenesis, and the progression and recurrence factors of tumors. The heterogeneity, treatment resistance, and immune evasion exhibited by tumors pose significant challenges to their early diagnosis and treatment, thus representing major obstacles in the medical advancements (4–7). The intricate tumor microenvironment is pivotal in tumor progression, with various signaling pathways contributing to tumor promotion (8). In addition, the limited delivery of small-molecule chemotherapeutic agents to tumor cells often results in suboptimal therapeutic outcomes (9, 10). Consequently, extensive researches have been conducted to develop effective anti-tumor drugs.

Currently, a variety of anti-tumor agents, including conventional chemotherapeutics, molecular targeted therapies, and immunotherapeutic drugs, have been introduced into clinical practice. Despite significant advancements in cancer pharmacotherapy, numerous challenges remain, including the toxic and adverse effects of chemotherapeutic agents (11), the emergence of tumor drug resistance (12), and poor response to immunotherapies (13). The ongoing advancements in nanotechnology not only have enabled nanomaterials to achieve high sensitivity and specificity in tumor detection, but also facilitate targeted drug delivery to tumor cells, thereby presenting extensive potential applications in tumor diagnosis and treatment (14–16). This review will concentrate on the application of DNA nanomaterials in tumor diagnosis and therapy.

The concept of DNA nanostructures was initially introduced by Professor Seeman and his colleagues. Over the past four decades, DNA nanotechnology has achieved significant breakthroughs (17). DNA, a biological macromolecule, serves as a fundamental carrier for the storage and transmission of genetic information. According to the Watson-Crick base pairing principle, single-stranded DNA can hybridize to form a double-stranded structure. Consequently, with precise base design, linear DNA can be engineered to form nanoscale three-dimensional structures (18, 19). The DNA assembly process is relatively simple and can be quickly assembled into the desired structure through an annealing process. The key to DNA nanostructures assembly is the precise design of single-strand sequences and the stability of dynamics and thermodynamics during the assembly process (20). In comparison to other nanomaterials, the advantages of DNA nanomaterials in the field of biomedicine: (1) Superior biocompatibility: DNA nanomaterials exhibit excellent biocompatibility with low immunogenicity, enabling both *in vivo* and *in vitro* applications while minimizing adverse effects and immune rejection. (2) High programmability: DNA can self-assemble into diverse nanostructures with precise shapes and functions through efficient and well-defined design protocols. (3) Enhanced structural stability: DNA nanostructures demonstrate significantly improved resistance to nuclease degradation, facilitating stable *in vivo* performance. (4) Efficient cellular internalization and tissue penetration: Tetrahedral framework nucleic acids can autonomously enter cells and penetrate whole tissues. (5) Versatile functionalization capacity: DNA nanostructures can be modified with various molecules (e.g., aptamers, drugs) for applications in drug delivery, bioimaging, and biosensing.These characteristics make DNA nanostructures promising candidates for drug delivery applications. Researchers have developed a variety of DNA-based nanomaterials characterized by predictability, controllable shape and size, topological complexity, and the potential for surface chemical modification and functionalization.

## 2 DNA nanostructures

**Figure 1:**
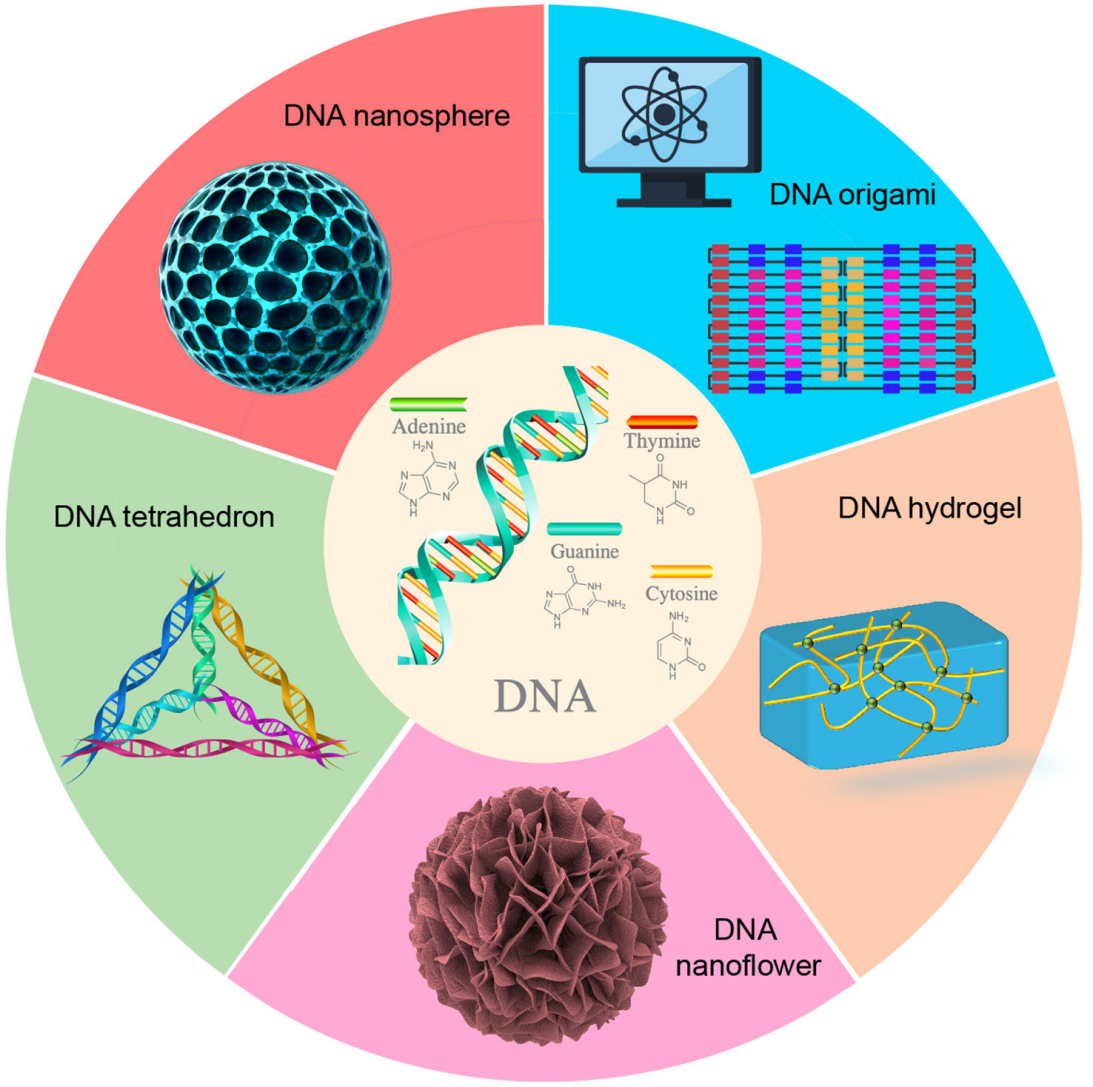
Common DNA nanostructures.

DNA represents a highly programmable material capable of being assembled into a variety of nanostructures through sequence-specific complementary hybridization, including DNA origami, DNA tetrahedrons, DNA nanoflowers, DNA hydrogels, and DNA nanospheres (**Figure 1**, **Table 1**). The size and morphology of these DNA nanostructures can significantly influence their efficiency in cellular internalization. Currently, advanced computational tools have been developed to facilitate the design and analysis of DNA nanostructures (21, 22), enabling researchers to tailor these structures to specific requirements.

**Table 1:** The types and key features of DNA nanostructure.

Type	Key features	References
DNA Origami	dual-mode imaging	(23–31)
DNA Tetrahedra	self-assembled nucleic acid frame	(32–42)
DNA nanoflowers	irregular self-assembly	(43–48)
DNA hydrogel	DNA hybridization rolling circle amplification	(49–63)
DNA nanospheres	rolling circle amplification	(64, 65)

DNA origami is constructed by folding a long single-stranded “scaffold” DNA into a predetermined shape, utilizing a set of short synthetic strands as “staples” to stabilize the overall structure. Consequently, DNA origami exhibits high programmability, spatial addressability, and structural complexity, making it extensively applicable in biosensing, biological imaging, biological computing, and biomedicine (23–27). Furthermore, DNA origami possesses multiple properties, such as flexibility, elasticity, plasticity, and mechanical stability, endowing it with significant potential for applications in biomechanical and physicochemical domains (28). The self-assembly of DNA origami presents numerous significant advantages. Firstly, each base of the DNA strand within a DNA origami structure can be modified, allowing for precise molecular-level manipulation (29, 30). Secondly, the self-assembly process exhibits a high degree of parallelism, enabling the simultaneous folding of billions of DNA origami structures within just a few microliters of volume. Thirdly, the capacity of DNA to bind with various molecules facilitates its use not only in biological labeling and detection, but also as a carrier for drug delivery (31). Through DNA origami technology, it is possible to construct two-dimensional and three-dimensional structures at the nanometer scale, which plays a crucial role in cancer treatment.

DNA tetrahedrons represent another prevalent DNA nanostructures with substantial potential applications in the biomedical field (32). Prior studies have demonstrated that DNA tetrahedrons, as self-assembled nucleic acid materials, are instrumental in antibacterial therapy (33, 34), tissue regeneration (35–37), treatment of nervous system diseases (38–40), osteoarthritis treatment (41), and tumor therapy (42–44). Additionally, DNA nanoflowers represent a class of multifunctional nanostructures characterized by the irregular self-assembly of DNA. These structures have found extensive applications in biosensing, biological imaging, and therapeutic interventions. Through the specific design of DNA nanoflowers, researchers have achieved highly sensitive detection of Staphylococcus aureus (45) and various mycotoxins (46). In terms of Alzheimer’s disease, DNA nanoflowers have been engineered to penetrate the brain and target neurons, facilitating the delivery of miR-124 and rutin, thereby exerting a synergistic therapeutic effect (47). Furthermore, by incorporating a diverse array of aptamers and peptides into DNA nanoflowers, researchers have demonstrated its ability to bind to cancer cells that overexpress HER2, highlighting significant potential for tumor diagnosis and treatment (48).

In addition, DNA can be engineered into hydrogel structures. In 1996, Nagahara and Matsuda pioneered the design and construction of the first DNA hydrogel, which formed a gel network polymer through DNA hybridization (49). Subsequently, researchers also developed DNA crosslinking (50, 51) and enzyme polymerization (52) for the synthesis of DNA hydrogels. Furthermore, the synthesis of DNA hydrogels via rolling circle amplification (RCA) is recognized as an efficient approach for the cost-effective and large-scale production of DNA materials (53–55). DNA hydrogels possess the predictable structure, adjustable mechanical strength, and numerous binding sites, exhibiting excellent biocompatibility and stability. These characteristics render them highly promising as scaffold materials for the applications in disease diagnosis (56, 57), regulation of tissue regeneration (58–60), and drug delivery (61–63). DNA nanospheres are also prevalent nanostructures. Researchers have developed DNA nanospheres activated by rolling circle amplification products and functional hairpins, which facilitate the delivery of the antitumor drug doxorubicin (DOX) for biological imaging and tumor therapy (64). Additionally, researchers can assemble DNA molecules into dendritic structures, characterized by DNA clusters emanating from a single branching point. These functional dendritic structures can interact with scavenger receptors on the cell surface, mediate rapid endocytosis, and play a crucial role in the intracellular transport of biomolecules, including small molecules, DNA/RNA, peptides, and proteins (65).

## 3 Screening and application of nucleic acid aptamer

**Figure 2:**
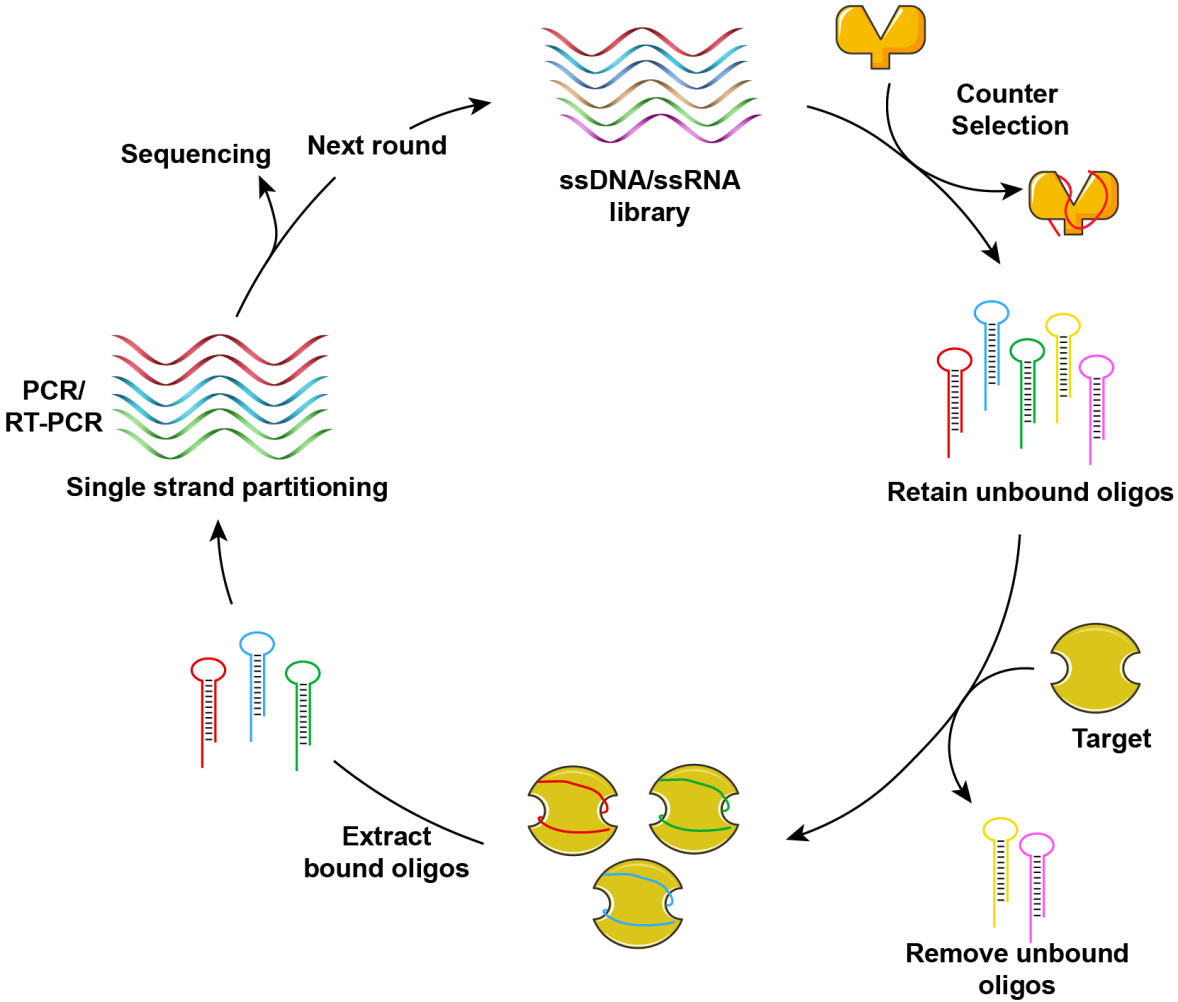
Schematic diagram of screening nucleic acid aptamers by SELEX.

Nucleic acid aptamers represent a distinct class of functional nucleic acids, consisting of single-stranded DNA or RNA molecules. These aptamers can fold into unique tertiary structures, exhibiting high specificity and affinity for their respective targets (66). The initial screening of oligonucleotide aptamers was accomplished using the SELEX (Systematic Evolution of Ligands by Exponential Enrichment) technology in 1990 (67–69). **Figure 2** illustrates the fundamental process of aptamer screening via SELEX technology. Through multiple rounds of cyclic screening (ranging from several to dozens) from a comprehensive DNA or RNA library, aptamers exhibiting specificity, affinity, and stability are isolated. The nucleic acid aptamers identified through this process are often referred to as “chemical antibodies”, possessing specificity and affinity comparable to antibodies. In contrast to antibodies, nucleic acid aptamers offer several structural and functional advantages, including ease of chemical synthesis and modification, programmability, and low immunogenicity. These aptamers demonstrate a broad range of target recognition capabilities, encompassing metal ions (70–73), small molecules (74–77), proteins (78, 79), and tumor markers (80–83). Aptamers possess significant potential for diverse applications within the field of biomedicine. This section specifically addresses the potential applications of nucleic acid aptamers in terms of tumor.

**Figure 3:**
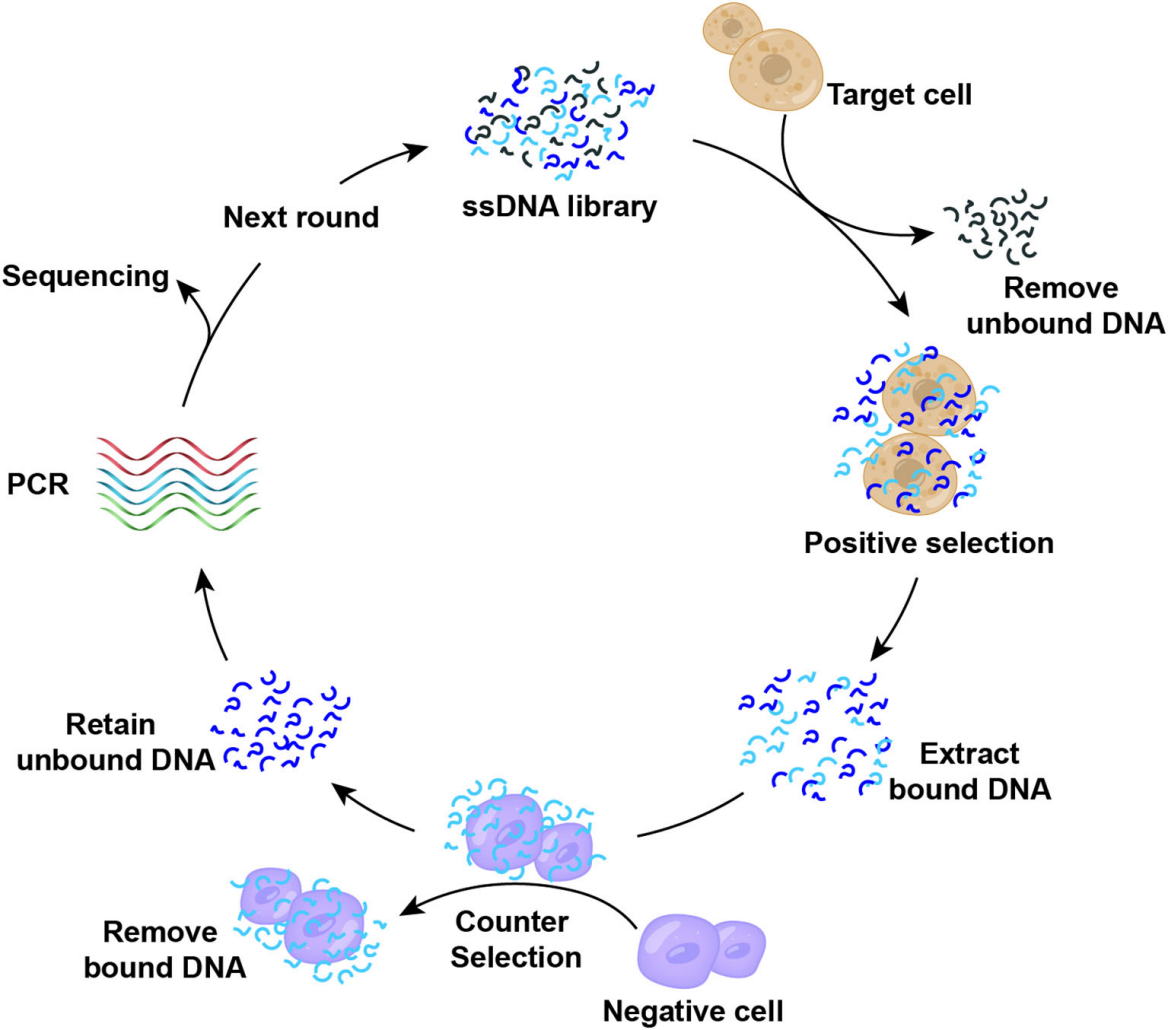
Cell-SELEX screening of nucleic acid aptamers.

Building upon the traditional SELEX method for screening nucleic acid aptamers, researchers have developed the Cell-SELEX technology (84). **Figure 3** illustrates the fundamental process of isolating cell-specific aptamers using Cell-SELEX method. This technology enables the identification of nucleic acid aptamers with high selectivity and specificity for binding to target cells through the interaction between specific cells and a nucleic acid library, including positive screening, reverse screening, cyclic screening, and sequencing. The Cell-SELEX is extensively utilized in tumor diagnosis and treatment. Primarily, it allows for the screening of aptamers with high selectivity for recognizing specific tumor cells (85–88), thereby establishing a foundation for subsequent applications in terms of tumor. Furthermore, the Cell-SELEX-based aptamer screening method facilitates the discovery of biomarkers associated with tumor cells (89, 90). Furthermore, we introduced the aptamers and their corresponding targets (**Table 2**). For instance, researchers have identified the aptamer spl3c, which can specifically recognize cytoskeleton-associated protein 4 (CKAP4) in bladder cancer cells. CKAP4 is significantly associated with the metastasis and poor prognosis in bladder cancer, suggesting that the spl3c aptamer holds potential for tumor monitoring applications in future (91). Furthermore, by employing metastatic colorectal cancer cells as target cells and non-metastatic cells as controls, it was discovered that the W3 aptamer can effectively differentiate between cancer cells with varying metastatic potentials. The W3 probe can also be utilized to capture W3-positive circulating tumor cells from patients (92). Consequently, the application of cell screening strategies enhances the specificity and efficiency of the development of tumor biomarkers. The Cell-SELEX screening has also been applied to investigate the drug resistance in tumor cells. Researchers have utilized multidrug-resistant hepatocellular carcinoma (HCC) cells to screen for specific aptamers, ultimately identifying the PS-ZL-7c aptamer, which exhibits high selectivity to drug-resistant tumor cells. This finding indicates their potential use in the precise identification of multidrug-resistant hepatocellular carcinoma (93).

**Table 2:** The aptamers and corresponding targets.

Aptamer	Targets	References
spl3c	cytoskeleton-associated protein 4 (CKAP4)	(91)
W3	W3-positive circulating tumor cells	(92)
PS-ZL-7c	multidrug-resistant hepatocellular carcinoma cells	(93)
AP-9R	lung cancer stem cells	(94)
PL1	PD-L1	(95)

Furthermore, the researchers identified that certain aptamers possess intrinsic tumor therapeutic properties, referred to as therapeutic aptamers. Through cell screening, the aptamer AP-9R was isolated, which exhibits high affinity for lung cancer stem cells. Its specific recognition target is the Ca^2+^-dependent membrane-binding protein Annexin A2, whose expression is closely associated with tumor stemness, leading to metastasis and poor clinical prognosis. Notably, the AP-9R aptamer can antagonize the expression of Annexin A2, thereby inhibiting tumor progression (94). Additionally, it has been observed that tumor cells can evade immune surveillance by expressing PD-L1, which binds to PD-1 on T cells, thereby inhibiting their function. By targeting cells expressing PD-L1, researchers employed the Cell-SELEX method to screen for PL1 aptamers, which specifically bind to PD-L1 and inhibit the PD-1/PD-L1 interaction. This inhibition restores the function of T cells suppressed by the PD-1/PD-L1 axis, thereby exerting an anti-tumor effect. Consequently, PL1 aptamers hold potential as alternative therapeutic agents in tumor immunotherapy (95).

Aptamers are incorporated into nanostructures to develop functionalized nanomaterials, which possess extensive applications in biological imaging and disease treatment. For instance, by constructing a network of DNA scaffolds modified with nucleic acid aptamers on the surface of living cells, it is possible to encapsulate cells under biocompatible conditions and manipulate cell-cell interactions to endow cells with additional functionalities (96). To address the instability of aptamers *in vivo*, their resistance to serum nucleases can be enhanced through covalent chemical modifications, thereby improving their stability and targeted recognition (97). Moreover, the application of monovalent aptamers in tumor targeting faces challenges, primarily due to their limited recognition capabilities and the absence of single receptor-mediated internalization. To overcome these limitations, researchers have developed a circular aptamer with dual-targeted ability of simultaneously recognizing two distinct biomarkers on living cells. This advancement results in improved recognition and internalization, demonstrating exceptional selectivity for tumor cells (98).

## 4 The function of DNA nanomaterials in regulating cellular biological processes

DNA nanomaterials have the potential to modulate cellular biological processes. Researchers have integrated the proton-driven assembly of DNA nanostructures with lysosome-mediated endocytosis to modulate lysosomal functions within cells, a process termed “lysosomal interference” (99). This approach can impede the degradation of nucleic acid drugs within lysosomes, thereby enhancing gene silencing efficacy and augmenting the role of nanomedicines within cells. To achieve precise regulation of intracellular mitochondrial function, researchers have developed an intracellular K^+^-mediated dynamic assembly of DNA tetrahedrons. This innovation effectively regulates mitochondrial energy metabolism in living cells, significantly inhibiting mitochondrial aerobic respiration and glycolysis, thereby reducing intracellular adenosine triphosphate (ATP) production and markedly inhibiting cell migration (100). The DNA-based dynamic assembly system can exploit the acidic microenvironment of lysosomes to specifically induce the formation of hydrogel structures within living cells. This strategy has the potential to modulate cellular behavior, encompassing cytoskeletal deformation, the retraction of cellular projections, and the facilitation of cell migration (101). To address the challenges in the acquisition and culture of stem cells, researchers have devised a DNA network efficiently capturing bone marrow mesenchymal stem cells (BMSCs) and providing an optimal microenvironment for cell culture to sustain normal cellular activity, which offers an effective method for stem cell capture (102). Furthermore, researchers have engineered dynamic, light-controlled reversible DNA nanostructures that enable precise regulation of cellular biological processes (103).

## 5 Application of DNA nanostructures in tumor diagnosis

Currently, tumor diagnosis primarily relies on pathological tissue biopsy and imaging, including magnetic resonance imaging (MRI), computed tomography (CT), ultrasound, and X-ray examination. Additionally, the biomarkers, such as oncofetal proteins, tumor-associated antigens, enzymes, and hormones, can be detected in the blood, urine, bile, and cerebrospinal fluid of tumor patients. With further research on transcriptomics, some molecules, including microRNAs (miRNAs), long non-coding RNAs (lncRNAs), circular RNAs (circRNAs), and circulating DNA fragments, can serve as the biomarkers for tumor diagnosis. Notably, the regulatory role of miRNA expression is pivotal in tumor development and progression, functioning as oncogenes or tumor suppressor genes to facilitate or inhibit tumor growth. Consequently, miRNAs hold potential as biomarkers for tumor diagnosis.

The development of highly sensitive miRNA detection methods not only enhances early cancer diagnosis but also provides critical insights for therapeutic decision-making across various stages of cancer treatment. To achieve high-sensitivity detection of tumor-associated miRNAs, researchers have employed a sustainable cascade catalytic hairpin assembly amplification strategy, resulting in significant signal amplification and enabling the detection of miRNA-155 in tumor cells with high sensitivity (104). Additionally, the research team led by Park HG has developed an ultrasensitive method for miRNA detection utilizing target-induced chain amplification (CAR), which successfully achieves precise detection of specific miRNAs down to a single copy level in tumor cells (105). Furthermore, the DNA tetrahedron, activated through *in situ* catalytic hairpin assembly, functions as an electrochemical biosensor. Upon encountering the tumor biomarker miRNA-141, an enzyme-free cycle is initiated, resulting in an amplified electrochemical signal. This process significantly reduces the detection time and enhances the sensitivity, facilitating the rapid and precise identification of tumor markers (106).

The regulatory mechanisms of miRNA are notably complex, as a single miRNA can regulate multiple mRNAs, and conversely, a single mRNA can be regulated by various miRNAs. Simultaneous detection of multiple miRNAs within living cells could yield substantial information, thereby enhancing the specificity and accuracy of disease diagnosis. To achieve concurrent detection of multiple targets, a three-dimensional DNA scaffold has been designed, which is easily prepared and exhibits high sensitivity, enabling the simultaneous detection of multiple low-abundance miRNAs in living cells (107). Furthermore, the researchers engineered a tetrahedral DNA framework capable of detecting a diverse array of intracellular miRNA with high sensitivity and selectivity. This framework allows for the modification of the DNA sequence as needed, facilitating the detection of other target genes within the cells. This adaptability in detection underscores the programmable nature of DNA sequences (108). Furthermore, by integrating the exponential rolling circle amplification method with the linear rolling circle amplification method, the researchers developed a circular symmetric nanostructure, which can hybridize with the specific miRNA on either side, significantly enhancing the sensitivity of miRNA detection (109). The DNA nanostructures, designed using logic operations, incorporates three recognition modules and three reporting modules, forming three “AND” logic gates. During operation, each “AND” logic component emits an “ON” signal in the presence of a bispecific miRNA, thereby enabling precise miRNA detection (110).

The identification of biomarkers associated with tumor metastasis is crucial for assessing the risk of metastatic progression. Consequently, researchers have developed an advanced DNA nanodevice incorporating an ATP-responsive aptamer sensor and an matrix metalloproteinase 2/9 (MMP2/9)-cleavable peptide-nucleic acid copolymer, which is engineered to sequentially interact with metastasis-related targets, MMP2/9 and ATP, within the extracellular tumor microenvironment, thereby enabling the concurrent detection of multiple biomarkers that promote tumor metastasis (111). Proteases, overexpressed in cancer cells, have been recognized as the biomarkers of malignant tumors. The researchers designed the nucleic acid-peptide-nucleic acid copolymer to stabilize the aptamer structure, allowing the protease to selectively activate the aptamer probe and generate tumor-specific fluorescence signals *in vivo*, thereby achieving precise molecular detection (112).

Furthermore, exosomes present a promising potential as biomarkers for non-invasive cancer diagnosis. However, achieving high sensitivity and accuracy detection remains a significant challenge. To address this, the researchers engineered a nucleic acid AND logic gate sensor utilizing dual aptamers to enable the concurrent detection of two tumor-associated proteins: protein tyrosine kinase 7 (PTK7) and prostate-specific membrane antigen (PSMA) in exocrine T lymphocytes from human acute lymphoblastic leukemia, which enhances the detection accuracy and holds potential for application in tumor diagnosis (113). For the detection of the breast cancer biomarker HER2, the researchers developed a light-responsive DNA origami structure, which incorporated a protein coating with targeted function on its surface and added a serum albumin camouflage agent to protect the DNA nanostructures. Upon light irradiation, the material undergoes cleavage, triggering the release of the camouflage protein, thereby allowing the targeting moiety to specifically recognize HER2 (114).

In the early stages of tumorigenesis, tumor cells and DNA can be released into peripheral blood, forming circulating tumor DNA (ctDNA). CtDNA has emerged as a novel biomarker in liquid biopsy, offering significant implications for early tumor diagnosis and recurrence monitoring. Detecting ctDNA in serum with high sensitivity remains a significant challenge. Researchers have employed DNA-rN1-DNA-mediated surface-enhanced Raman scattering to successfully identify base pair-mutated ctDNA from normal genes in lung cancer, enhancing the signal amplification. Then, the sensitivity testing of ctDNA was conducted on serum samples from lung cancer patients (115). By integrating tetrahedral DNA nanostructures with carbon nanotubes, the detection of ctDNA associated with AKT2 genes in triple-negative breast cancer has been achieved, and this platform also facilitates the identification of other biomarkers (116). Furthermore, the biocompatibility of erythrocytes was utilized to adhere tetrahedral DNA nanostructures, enabling the identification of specific targets by the tetrahedrons, with methyl blue serving as a signal probe for the efficient and specific detection of ctDNA (117). Recent studies have successfully engineered a self-assembled photocatalytic DNA nanoflower system, which enables high-sensitivity and low-cost quantitative detection of carcinoembryonic antigen (CEA), demonstrating significant potential for clinical cancer diagnostics (118). Furthermore, circulating tumor cells (CTCs) have emerged as promising liquid biopsy biomarkers for cancer screening and early detection. Researchers have developed self-assembled DNA nanomachines functionalized with aptamer-based recognition probes, achieving highly sensitive CTC detection (119).

## 6 Application of DNA nanostructures in tumor therapy

DNA nanostructures have been extensively investigated as platforms for drug delivery. Given the inherently negative charge of cell membranes, DNA nanostructures can enter cells via endocytosis, subsequently delivering DNA carriers and drugs to lysosomes for drug release. The integration of aptamers with various DNA nanostructures represents a prevalent strategy in tumor therapy (120). The utilization of DNA nanotechnology in drug delivery and therapeutic applications holds substantial significance.

### 6.1 Chemotherapy

Chemotherapy remains the predominant modality in cancer treatment, extensively employed across diverse cancer types. Nonetheless, traditional chemotherapeutic agents lack intrinsic tumor cell specificity, leading to suboptimal drug concentrations at the tumor sites. This limitation not only diminishes therapeutic efficacy but also inflicts damage on healthy tissues, accompanied by adverse side effects. DNA nanostructures, characterized by high biocompatibility and modifiability, serve as effective drug delivery vehicles, which exhibit enhanced permeability and retention effects, enabling accumulation at tumor sites. When modified with aptamers, they achieve targeted delivery, facilitating precise drug administration. For instance, the widely used broad-spectrum antitumor drug DOX can intercalate into the base pairs of double-stranded DNA, enabling drug loading within DNA nanostructures and augmenting their anti-tumor efficacy as a nano-drug delivery system (121–124). Additionally, researchers have developed an AS1411 aptamer-modified DNA nanomaterial loaded with the antineoplastic drug 5-fluorouracil (5-FU), creating a tumor-targeting nanodrug. This innovation addresses the limitations of conventional chemotherapeutic agents, which often lack specific targeting capabilities, thereby enhancing the therapeutic efficacy against breast cancer (125). By conjugating antitumor drugs with monoclonal antibodies, DNA wireframe cubes facilitate precise control over the drug-to-antibody ratio, optimizing their role in anti-tumor therapy (126). Furthermore, to address challenges such as poor water solubility, low bioavailability, and inadequate drug accumulation associated with chemotherapeutic agents, the researchers have devised a novel DNA nanocarrier, which encapsulates erlotinib, serves as an effective antitumor drug delivery mechanism with a pronounced inhibitory effect on non-small cell lung cancer (127).

The chemotherapy resistance remains a significant barrier to effective tumor treatment, leading to treatment failures, prolonged hospital stays, and increased patient mortality. To address chemotherapy resistance, researchers can enhance therapeutic efficacy and overcome resistance by designing DNA nanostructures that target specific organelles, which holds significant practical implications for tumor therapy (128). Cisplatin, a first-line chemotherapeutic agent, primarily encounters resistance due to reduced intracellular drug accumulation. By developing DNA nanostructures as efficient drug carriers, the cellular uptake is markedly increased, thereby enhancing their antitumor efficacy (129). Furthermore, researchers have engineered DNA nano-scaffolds in conjunction with 5-fluoro-2-deoxyuridine. These drug-loaded nanomaterials can mitigate the low sensitivity of colorectal cancer cells to 5-FU, resulting in increased cytotoxicity and apoptosis induction (130).

### 6.2 Gene therapy

Gene therapy involves the introduction of exogenous genes into target cells via vectors, aiming to treat diseases by altering the expression of intracellular genes. Gene therapy has emerged as a novel approach for the treatment of malignant tumors. Numerous clinical trials of gene therapy for tumors are being conducted globally, and several products have received approval. While viral vectors are predominant in gene delivery and have been extensively studied, there is concern among researchers regarding the potential for viral vectors to elicit antiviral immune responses, which may result in adverse reactions. The rapid advancement of nanotechnology, particularly the ongoing development of DNA nanomaterial vectors, target genes can be delivered to tumor cells while minimizing the immunogenicity associated with the material itself.

RNA interference (RNAi) is a molecular biological mechanism that employs non-coding nucleic acids to modulate gene expression at the post-transcriptional level, utilizing agents such as small interfering RNA (siRNA) or short hairpin RNA (shRNA) to disrupt the expression of target genes. In a recent study, the design of a nano-cage on tetrahedral DNA encapsulating therapeutic siRNA demonstrated effective endosomal escape and down-regulation of epidermal growth factor receptor (EGFR) expression in A549 tumor cells, thereby significantly inhibiting tumor growth (131). Antisense oligodeoxynucleotides (ASOs) inhibit or degrade RNA translation by specifically targeting RNA sequences. These ASOs can be employed to adsorb and eliminate oncogenic miRNAs or silence aberrantly expressed miRNAs within cells, serving as a therapeutic approach in anti-tumor chemotherapy. Utilizing this strategy, researchers have developed self-assembled DNA nanospheres designed for the intracellular adsorption and clearance of miRNA-21. These nanospheres deliver a substantial quantity of repetitive ASOs, effectively capturing miRNA-21, inducing alterations in associated signaling pathways, and synergizing with DOX to promote apoptosis in tumor cells (132). Furthermore, DNA nanostructures incorporating therapeutic ASOs targeting c-raf-1 mRNA, as well as DNase targeting MMP-9, have been engineered to collectively inhibit the proliferation and migration in A549 cells (133, 134). Gene editing also represents a crucial modality in gene therapy. Among the various gene editing technologies, CRISPR/Cas9 is widely regarded as the most precise. Researchers have developed a proton-activated DNA nanosystem that modulates gene expression by integrating Cas9/sgRNA with DNAzyme, thereby enhancing the therapeutic efficacy in breast cancer (135).

### 6.3 Immunotherapy

In recent years, immunotherapy has emerged as a highly promising approach for cancer treatment, opening up new pathways for the treatment of cancer. The increasing elucidation of immune response mechanisms in tumorigenesis presents both opportunities and challenges for immunotherapy. Notably, immune checkpoint blockade (ICB) therapy has been considered as a novel paradigm in cancer treatment (136). Evidence indicates that the efficacy of immunotherapy in cancer patients is significantly influenced by the tumor microenvironment (137, 138). Despite the growing research on tumor immunotherapy, daunting challenges remain due to the complexity of the tumor microenvironment and immunosuppressive effects. These challenges include low drug delivery efficiency, variable patient response rates, and immune-related adverse reactions. With the ongoing advancements in nanotechnology, nanomaterials have found extensive applications in tumor immunotherapy.

Functional DNA nanomaterials, known for their excellent biosafety, serve as effective drug carriers for targeted delivery, thereby demonstrating significant potential in tumor immunotherapy. Researchers have engineered a pH-responsive DNA origami nanodevice capable of efficiently delivering tumor antigen peptides to antigen-presenting cells, thereby enhancing anti-tumor immunity (139). The efficient and high-purity isolation of immune cells with minimal damage is crucial in the field of immunotherapy. Consequently, researchers have developed a precisely controlled DNA network, incorporating multifunctional modules for cell capture and immune adjuvant activities, to specifically isolate and culture T lymphocytes *in situ*. The DNA network achieved a 98% purity in capturing tumor-infiltrating T cells, with 90% survival rate, which demonstrated a significant therapeutic effect in tumor immunotherapy (140).

To modulate the anti-tumor effects of immune cells, researchers have engineered a DNA nanostructure targeting tumor-associated macrophage lysosomes. This nanostructure can be delivered to phagocytic lysosomes via receptor-mediated endocytosis, where it specifically inhibits cysteine protease activity, enhances antigen presentation capabilities, and facilitates tumor cell eradication through the activation of CD8+ T cells. These findings demonstrate that DNA nanostructures can be precisely reprogrammed at the organelle level to achieve immune modulation (141). Furthermore, tetrahedral DNA nanostructures are capable of actively entering macrophages, promoting the activation of the STING (Stimulator of Interferon Genes) signaling pathway and M1 polarization, and increasing the expression of IFN-β and iNOS, thereby initiating macrophage activation and anti-tumor responses both *in vitro* and *in vivo* (142). Regarding immune cell activation, the researchers have developed DNA line-polymerized nanospheres that can deliver multivalent non-methylated CpG, which can continuously stimulate Toll-like receptor 9 on immune cells, thereby significantly enhancing immune cell activation and further inducing tumor cell apoptosis (143). Furthermore, functional DNA nanomaterials hold potential for enhancing cancer immunotherapy through the delivery of tumor vaccines. This involves the incorporation of novel tumor antigens into vaccines, followed by the construction of DNA-RNA nanocapsules to serve as carriers for these antigens, realizing potent anti-tumor immunotherapy (144).

### 6.4 Photodynamic therapy

Photodynamic therapy (PDT) represents an innovative approach to tumor treatment, wherein a photosensitizer is administered and subsequently activated by a specific wavelength of laser light upon reaching the tumor tissue, thereby inducing cytotoxic effects on tumor cells. DNA nanostructures facilitate the precise and efficient delivery of photosensitizers to tumor sites, thereby augmenting the efficacy of PDT. For instance, researchers have employed triangular DNA origami as nanocarriers to deliver the carbazole derivative photosensitizer BMEPC to MCF-7 breast cancer cells, which can be activated by near-infrared light, initiating a photodynamic reaction (145). Additionally, DNA nanocapsules enables effective loading and delivery of photosensitizers, allowing for precise tumor targeting and enhanced photodynamic efficacy (146).

### 6.5 Combination therapy

During the course of tumor treatment, the integration of multiple therapeutic modalities has garnered increasing attention due to its superiority over monotherapy, with many demonstrating significant antitumor efficacy. The ongoing advancements in nanotechnology have underscored the pivotal role of DNA nanomaterials in combined tumor therapies. Specifically, the chemical-gene synergistic therapy model utilizing DNA nanomaterials has exhibited enhanced antitumor effects. For instance, pH-responsive multifunctional DNA nanomaterials have been engineered as carriers for the controlled release of DOX and anaplastic lymphoma kinase (ALK)-specific siRNA, with low toxicity and commendable biocompatibility, thereby contributing effectively to tumor suppression (147). DNA self-assembly facilitates the construction of DNA nanospheres with precise size control, which are concurrently loaded with the chemotherapeutic agent DOX and two distinct siRNAs (ALK-siRNA and Sur-siRNA). This approach culminates in the successful development of a multifunctional nanodrug delivery system, demonstrating exceptional efficacy in tumor chemical-gene therapy (148).

Furthermore, DNA nanomaterials designed for chemotherapy and photodynamic therapy have demonstrated the ability to specifically target tumor cells for the delivery of DOX and photosensitizers, thereby exerting a synergistic antitumor effect (149). DNA tetrahedral nanoplatform carrying nucleic acid drugs and photodynamic agents can effectively penetrate the cell membrane, subsequently transport the therapeutic agents to the mitochondria and nuclei of target cells, which facilitates a combined therapeutic approach involving gene therapy and photodynamic therapy, ultimately inducing apoptosis in tumor cells (150). Additionally, in term of combined chemotherapy and immunotherapy, researchers have developed tetrahedral DNA nanostructures capable of delivering platinum-based chemotherapeutic drugs and immune stimulators, which provide highly cytotoxic agents while activating the immune response via the STING pathway, demonstrating significant antitumor effects in the treatment of breast cancer (151).

Notably, the programmable nature of DNA nanomaterials enables their integration into multimodal diagnosis and treatment platforms. These innovative systems synergize diagnostic capabilities with therapeutic functions through the modular design of all-in-one nanostructures carrying both detection probes and therapeutic payloads. Early detection through cancer-associated biomarkers and targeted therapeutic delivery are critical for timely diagnosis and disease management. Integrated platforms combining both diagnostic and therapeutic functions represent a promising strategy for precision medicine, particularly in managing intractable diseases. The researcher developed a dual-stimuli-responsive DNA nanoframeworks activated by endogenous tumor-overexpressed enzymes APE1 and RNase H, serving as a simultaneous detection and high-efficiency gene therapy platform for pancreatic cancer (152). This integration represents a paradigm shift toward precision oncology.

## 7 The challenges of DNA nanomaterials in biological and clinical applications

Globally, only 14 systemic cancer nanodrugs have been approved for clinical use, the majority of which are liposomal formulations of small molecule chemotherapeutic drugs (153). While DNA nanomaterials address certain limitations of other materials and offer significant advantages in disease diagnosis and treatment, they encounter numerous challenges in clinical translation. The foremost issue in the clinical application of DNA nanomaterials is their biosafety *in vivo*. Currently, safety assessments of DNA nanomaterials are predominantly conducted at the animal level, with a notable lack of empirical validation in human patients. Another critical challenge is the stability of DNA nanomaterials. Although DNA structures remain stable in culture media during laboratory experiments, they must withstand nuclease-mediated degradation in the blood once introduced into the body. Consequently, enhancing the *in vivo* stability of DNA nanomaterials and extending their circulation time in the blood are essential for optimizing their therapeutic efficacy. The shape and topology of DNA nanostructures are crucial determinants of their stability (154). Additionally, a promising strategy to enhance the *in vivo* stability of DNA nanostructures involves conjugating nucleic acids with lipids, thereby mitigating rapid degradation within the body.

Furthermore, the mechanisms by which DNA nanomaterials entry into cells, including endocytosis and endosomal escape, remain insufficiently explored, thereby constraining their broader applicability. In the process of delivering siRNA for gene therapy, it is imperative to enhance the lysosomal escape capabilities of these nanostructures, which can be achieved through aptamer functionalization facilitating targeted delivery. The design of DNA nanostructures must carefully consider their size, ranging from 25 to 150 nm, which exhibit improved tissue permeability and retention effects (155). Additionally, various physiological barriers within the human body, such as glomerular filtration and the blood-brain barrier, present significant challenges to the targeted delivery of nanocarriers.

## 8 Conclusion and prospect

Looking ahead, we envision four key directions for DNA nanomaterials in biomedicine: (1) Applications of DNA nanostructures in immunotherapy, including adjuvant delivery, antigen presentation, and immune checkpoint blockade. (2) Breakthroughs in biomimetic design of DNA nanostructures, particularly their prospects for transmembrane transport regulation. (3) Stimuli-responsive systems represent a crucial developmental direction by resolving the permeability-retention paradox in nanodrug delivery. (4) Applications of intelligent DNA biosensor-based multiplexed noninvasive biomarker analysis in tumor diagnosis.

Nanotechnology offers numerous innovative approaches for advancing biomedicine, particularly in the areas of disease diagnosis and treatment. DNA nanostructures exhibit several advantageous properties, including excellent biocompatibility, programmability, and ease of synthesis, modification, and functionalization. These characteristics facilitate their modification with conventional nucleic acid probes, which have been extensively utilized in biosensors and biological imaging, thereby pioneering novel methodologies for tumor diagnosis. Additionally, through precise design and assembly, DNA nanostructures have been functionalized, leading to significant advancements in the field of targeted tumor therapy. Despite the existing challenges, the ongoing development of nanotechnology is expected to pave the way for the future clinical application of DNA nanomaterials.
